# Characterization of the complete mitogenome of the endangered freshwater fish *Gobiobotia naktongensis* from the Geum River in South Korea: evidence of stream connection with the Paleo-Huanghe

**DOI:** 10.1007/s13258-022-01265-6

**Published:** 2022-06-08

**Authors:** Keun-Sik Kim, Dong-Won Kang, Keun-Yong Kim, Jung Soo Heo, Ha-Yoon Song, Ju-Duk Yoon

**Affiliations:** 1 Restoration Research Team (Fishes/Amphibians & Reptiles), Research Center for Endangered Species, National Institute of Ecology, 23 Gowol-gil, Yeongyang-gun, Gyeongsangbuk-do 36531 Republic of Korea; 2 Department of Genetic Analysis, AquaGenTech Co., Ltd, 48300 Busan, Republic of Korea; 3 Inland Fisheries Research Institute, National Institute of Fisheries Science, 32762 Geumsan, Republic of Korea

**Keywords:** Endangered species, Paleo-Huanghe, *Gobiobotia naktongensis*, *Gobiobotia pappenheimi*, Geum River, Mitogenome, Phylogeny

## Abstract

**Background:**

The freshwater fish *Gobiobotia naktongensis* (Teleostei, Cypriniformes, and Gobionidae) is an endangered class I species whose population size has been greatly reduced.

**Objective:**

To successfully protect and restore the highly endangered freshwater fish *G. naktongensis* from the Geum River in South Korea.

**Methods:**

The mitogenome was characterized using the primer walking method with phylogenetic relationships.

**Results:**

The complete mitogenome of *G. naktongensis* Geum River was 16,607 bp, comprising 13 protein-coding genes, 2 ribosomal RNA genes, and 22 transfer RNA (tRNA) genes. Seventeen substitutions were found by comparing the tRNA regions between *G. naktongensis* Geum and Nakdong Rivers and *G. pappenheimi*; most were specific to *G. naktongensis* Nakdong River, with changes in their secondary structures. The comparison between *G. naktongensis* Geum River and *G. pappenheimi* revealed differences in the lengths of the D-loop and two tRNAs (tRNA^Arg^ and tRNA^Trp^) and the secondary structures in the TΨC-arm of tRNA^His^. In the phylogenetic tree, *G. naktongensis* Geum River did not cluster with its conspecific specimen from the Nakdong River in South Korea, but showed the closest relationship to *G. pappenheimi* in mainland China.

**Conclusions:**

Our results support the existence of the Paleo-Huanghe River connecting the Korean peninsula and mainland China, suggesting that *G. naktongensis* in the Geum River should be treated as a different evolutionarily significant unit separated from that in the Nakdong River. The complete mitogenome of *G. naktongensis* Geum River provides essential baseline data to establish strategies for its conservation and restoration.

## Introduction

The freshwater fish *Gobiobotia naktongensis* (Teleostei, Cypriniformes, Gobionidae) is a small species with a total length of 6–8 cm (www.fishbase.org, 2022). It was first reported in the Nakdong River system in South Korea and was classified as a novel species by Mori (1935). Since then, ecological studies by Jeon and Son (1983) and Choi (1985) have shown that its distribution extends to the Geum and Han River systems in South Korea, respectively. This species is endemic to the Korean peninsula and has been designated and protected as endangered class I species since 2005 by the Wildlife Protection Act of the Ministry of Environment in South Korea. It is also classified as a vulnerable species in the Red Data Book of Endangered Fishes in Korea (NIBR 2014). Its population size has been greatly reduced by large-scale river engineering projects, such as the Four Major Rivers Restoration Project (2009–2011). There have been no reports of *G. naktongensis* in the Geum River since the construction of large weirs in 2013 (MOE/NIE 2013–2018a, 2013–2018b; MOE/NIBR 2013–2018). However, after the complete reopening of the Sejong-weir at the Geum River in October 2018, *G. naktongensis* was found 200 m downstream of the weir in April 2019.

**Fig. 1:**
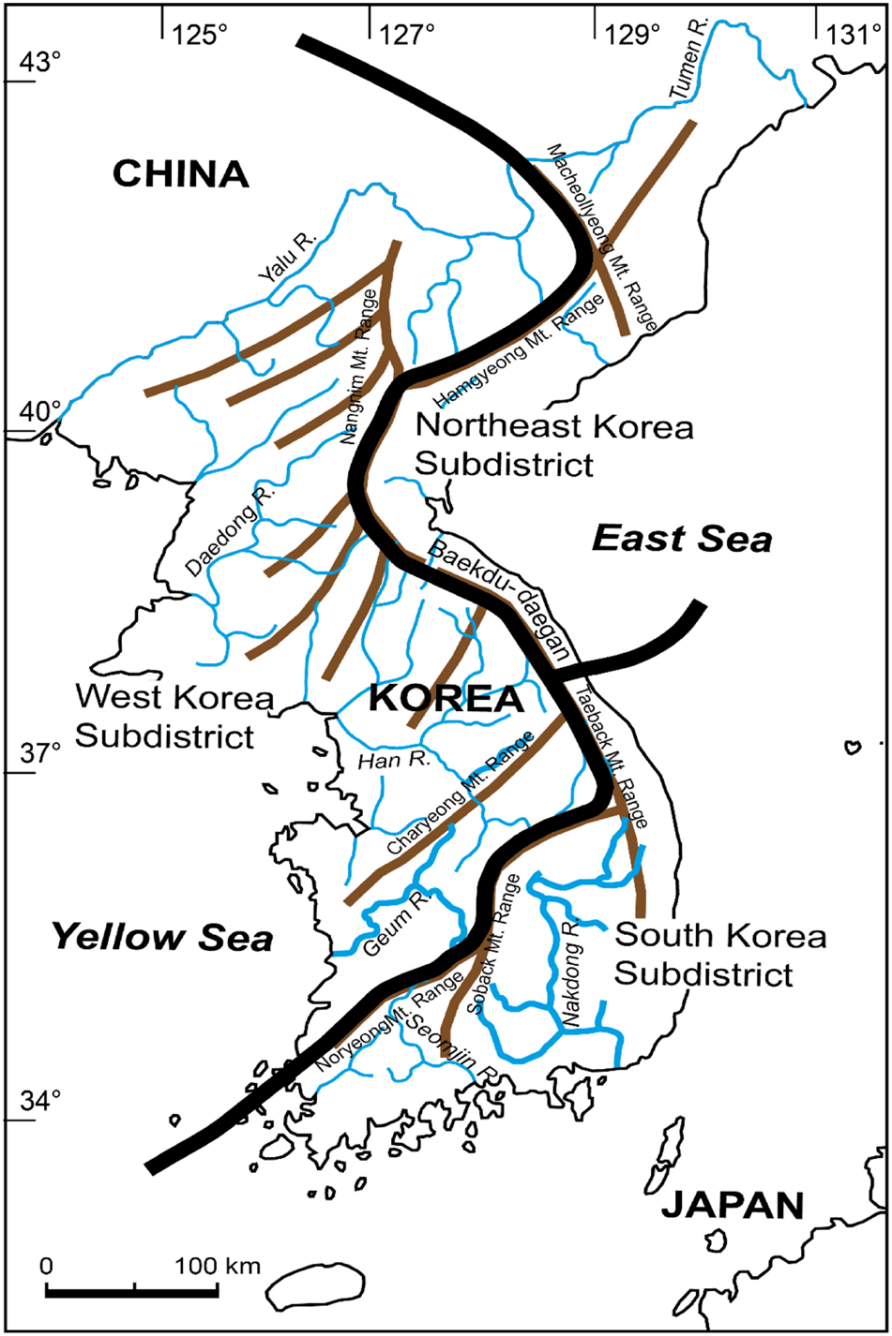
Major river systems in the Korean peninsula. The Geum and Nakdong River systems, in which two *Gobiobotia naktongensis* populations were reported, are indicated by thick lines. Three biogeographical areas based on freshwater fish assemblages were adopted from Kim et al. (2005)

To protect and restore endangered species successfully, it is necessary to establish effective strategies based on their population genetic structure. Mitochondrial genomes (mitogenomes) are powerful phylogenetic markers (Avise et al. 1987) or golden regions of DNA barcoding markers and are frequently used in ecological, evolutionary, and systematic studies, as well as in conservation studies of diverse vertebrate taxa. *G. naktongensis* is distributed in only four major river systems, the Nakdong, Geum, Han, and Imjin Rivers in South Korea. The Korean Peninsula is divided into three subdistricts based on the geological and biogeographical separation of freshwater fish fauna (Fig. 1) (Kim et al. 2005; Kim and Bang 2012). Given this biogeography, a high level of genetic variation is expected to exist between the *G. naktongensis* populations that inhabit different major river systems in the two different subdistricts, that is, the West Korea Subdistrict and South Korea Subdistrict. However, only one mitogenomic sequence of *G. naktongensis* is available for the Nakdong River (Hwang et al. 2013a) in the GenBank database (accession number KC353467).

The aim of this study was to analyze and characterize the complete mitogenome of *G. naktongensis* from the Geum River and to reveal the phylogenetic relationship by including two populations from different major river systems in South Korea.

## Materials and methods

Specimens of *G. naktongensis* were caught in 2020 from the Geum River in South Korea using a scoop net (mesh size: 4 × 4 mm). The captured individuals had a total length of 56 mm, a body length of 49 mm, and a weight of 1.09 g. They were anesthetized by submersion in an anesthetic agent (MS222; Aqualife TMS, Syndel Laboratories, Ltd., Canada). A small piece of the caudal fin was excised with sterile scissors, and the endangered fish was released after recovery from the anesthetic in clean water. All sampling was conducted with the permission of the Ministry of the Environment of Korea. Genomic DNA (gDNA) was extracted using TNES-urea buffer according to Asahida et al. (1996). The gDNA was stored in a voucher (NeF-00001) at the Research Center for Endangered Species. The mitogenome was divided into two regions and amplified by overlapping PCR amplification, according to Kim et al. (2012). The PCR products were sequenced by the primer walking method using 25 sequencing primers (available upon request) on a 3730*xl* DNA Analyzer (Applied Biosystems, Foster City, CA, USA). The sequence was deposited in GenBank under accession number MT539708.

The sequence data were assembled in a complete circular contig using DNA sequence analysis software (Sequencher 5.0; Gene Codes Corp., Ann Arbor, MI, USA). The mitogenomic sequence was annotated using the MITOS web server (Bernt et al. 2013) and MitoFish (Iwasaki et al. 2013) with those of other gobionid species publicly available in the GenBank database to determine the gene boundaries of protein-coding genes (PCGs) and ribosomal RNA (rRNA) genes. Transfer RNA (tRNA) genes were identified by tRNAscan-SE 1.21 (Lowe and Eddy 1997) to compare their secondary structures among *G. naktongensis* from Geum and Nakdong rivers in South Korea and *G. pappenheimi* from mainland China.

All mitochondrial genes, including PCGs, rRNA, and tRNA, were rearranged in the H-strand for further analysis. The identification of the exact start and stop codons of all PCGs was carried out after alignment using ClustalX 2.0 (Larkin et al. 2007) in MEGA-X software (Kumar et al. 2018). Nucleotide compositions were estimated and compared for all species in the genus *Gobiobotia* using the MEGA-X software (Kumar et al. 2018). To estimate the base composition bias, the strand asymmetry of the mitogenome of *G. naktongensis* Geum River was calculated using the following formulas: AT skew = [A − T]/[A + T] and GC skew = [G − C]/[G + C] (Perna and Kocher 1995). We also calculated the values of relative synonymous codon usage (RSCU) of the mitogenome of *G. naktongensis* Geum River using MEGA-X software (Kumar et al. 2018). The Ka/Ks ratio of 13 PCGs, excluding the stop codon of four *Gobiobotia* species, was calculated using DnaSP v5 (Librado and Rozas 2009).

The mitogenomic sequences of 77 species belonging to the family Gobionidae, including *G. naktongensis* Nakdong River, were retrieved from GenBank. They were aligned with the sequence of *G. naktongensis* Geum River in this study and manually refined for phylogenetic analysis. For phylogenetic analysis, the nucleotide matrix was partitioned into four groups, according to Inoue et al. (2005). The sequences of the 12 PCSs, excluding *nad6*, were divided according to codon position (i.e., the first and second positions of codon triplets), excluding the third codon position. Unambiguously aligned regions from 2 rRNA and 22 tRNA genes were obtained after eliminating divergent regions using Gblocks Server (http://molevol.cmima.csic.es/castresana/Gblocks_server.html) with default settings. Nucleotide matrices of 3617, 3617, 2527, and 1498 bp for the first and second codon positions of the PCSs, and rRNA and tRNA genes, respectively, were obtained. The alignment information is available upon request in the FASTA format.

Bayesian inference (BI) analysis was conducted using MrBayes 3.1.2 (Ronquist and Huelsenbeck 2003) for all representative species of the Gobionidae, including *G. naktongensis*. Two leuciscid species (*Leuciscus waleckii* and *Tribolodon hakonensis*) were used as outgroups. We selected the nucleotide substitution models that best fit each partitioned nucleotide matrix using jModelTest 2 (Darriba et al. 2012) based on the Bayesian information criterion (BIC). The general time-reverse (GTR) model, allowing invariant sites and a gamma distribution (the GTR + I + Γ model), was selected for all partitions. Four independent Markov chains were simultaneously used at 1,000,000 generations with sampling every 100 generations, and the first 25% was discarded as burn-in.

Maximum likelihood (ML) analysis was performed with RAxML 7.0.4 (Stamatakis 2006; Stamatakis et al. 2008). The concatenated nucleotide matrix was divided into four partitions. A RAxML search was executed for the best-scoring ML tree in a single program run (the “‐fa” option), instead of the default maximum parsimony starting tree. The best‐scoring ML tree of a thorough ML analysis was determined using the GTRGAMMAI model based on 200 inferences. Statistical support was evaluated using 1000 nonparametric bootstrap inferences. The resultant tree was illustrated using TreeView 1.6.6.

## Results and discussion

The complete mitogenome of *G. naktongensis* Geum River in South Korea is a circular molecule 16,607 bp in total length (Table 1), which is similar to the total length of other *Gobiobotia* species (16,609–16,637 bp; Table 2). It comprises 13 PCGs, two rRNA (12S and 16S rRNAs), 22 tRNA genes, and one control region (Table 1). Its gene content and order were identical not only to those of other congeneric species but also to those of other typical vertebrates. Twelve PCGs, excluding *nad6* and eight tRNA genes (tRNA^Gln^, tRNA^Ala^, tRNA^Asn^, tRNA^Cys^, tRNA^Tyr^, tRNA^Ser2^, tRNA^Glu^, and tRNA^Pro^), were positioned on the heavy strand (H-strand) and the origin of replication on the light strand (L-strand) (Hwang et al. 2013a; Kim et al. 2020; Kwak et al. 2021). Overlaps in sequences among the mitochondrial genes were found in ten genes with total of 27 bp and a range of 1–7 bp. The most prominent overlaps were detected between *atp8* and *atp6* and between *nd4L* and *nd4*, and the others were frequently found between PCGs and tRNA genes or between two adjoining tRNA genes. Although most PCGs started with ATG (a putative start codon), *cox1* started with GTG, the result of which is identical to that of most typical vertebrates (Tzeng et al. 1992; Miya et al. 2003; Shan et al. 2016). Ten out of 13 PCGs had complete stop codons (TAA or TAG), while the other three genes (*cox2*, *cox3*, and *cob*) had incomplete stop codons, such as T or TA. These incomplete stop codons can be converted to TAA by polyadenylation after transcription during mRNA maturation (Ojala et al. 1981).

**Table 1:** Gene compositions and positions of the mitogenome of *Gobiobotia naktongensis* from the Geum River in South Korea

Full gene name	Gene	Strand^a^	Positions	Size (bp)	Spacer (+)/ overlap (−)^b^	Start/stop codon	Anti- codons
tRNA-Phe	tRNA^Phe^	H	1–69	69	0		GAA
12S ribosomal RNA	12S rRNA	H	70–1031	962	0		
tRNA-Val	tRNA^Val^	H	1032–1103	72	0		TAC
16S ribosomal RNA	16S rRNA	H	1104–2791	1688	0		
tRNA-Leu2	tRNA^Leu2^	H	2792–2867	76	0		TAA
NADH dehydrogenase subunit 1	*nad1*	H	2868–3842	975	0	ATG/TAA	
tRNA-Ile	tRNA^Ile^	H	3846–3917	72	3		GAT
tRNA-Gln	tRNA^Gln^	L	3916–3986	71	‒ 2		TTG
tRNA-Met	tRNA^Met^	H	3988–4056	69	1		CAT
NADH dehydrogenase subunit 2	*nad2*	H	4057–5103	1047	0	ATG/TAA	
tRNA-Trp	tRNA^Trp^	H	5103–5173	71	‒ 1		TCA
tRNA-Ala	tRNA^Ala^	L	5176–5244	69	2		TGC
tRNA-Asn	tRNA^Asn^	L	5246–5318	73	1		GTT
Origin of light strand replication	OL	L	5319–5349	31	0		
tRNA-Cys	tRNA^Cys^	L	5350–5417	68	0		GCA
tRNA-Tyr	tRNA^Tyr^	L	5419–5489	71	1		GTA
Cytochrome *c* oxidase subunit I	*cox1*	H	5491–7041	1551	1	GTG/TAA	
tRNA-Ser2	tRNA^Ser2^	L	7042–7112	71	0		TGA
tRNA-Asp	tRNA^Asp^	H	7116–7187	72	3		GTC
Cytochrome *c* oxidase subunit II	*cox2*	H	7201–7891	691	13	ATG/T	
tRNA-Lys	tRNA^Lys^	H	7892–7967	76	0		TTT
ATP synthase F_0_ subunit 8	*atp8*	H	7969–8133	165	1	ATG/TAA	
ATP synthase F_0_ subunit 6	*atp6*	H	8127–8810	684	‒ 7	ATG/TAA	
Cytochrome *c* oxidase subunit III	*cox3*	H	8810–9594	785	‒ 1	ATG/TA	
tRNA-Gly	tRNA^Gly^	H	9594–9665	72	‒ 1		TCC
NADH dehydrogenase subunit 3	*nad3*	H	9666–10,016	351	0	ATG/TAG	
tRNA-Arg	tRNA^Arg^	H	10,015–10,083	69	‒ 2		TCG
NADH dehydrogenase subunit 4 L	*nad4L*	H	10,084–10,380	297	0	ATG/TAA	
NADH dehydrogenase subunit 4	*nad4*	H	10,374–11,756	1383	‒ 7	ATG/TAG	
tRNA-His	tRNA^His^	H	11,756–11,824	69	‒ 1		GTG
tRNA-Ser1	tRNA^Ser1^	H	11,825–11,893	69	0		GCT
tRNA-Leu1	tRNA^Leu1^	H	11,895–11,967	73	1		TAG
NADH dehydrogenase subunit 5	*nad5*	H	11,968–13,803	1836	0	ATG/TAA	
NADH dehydrogenase subunit 6	*nad6*	L	13,800–14,321	522	‒ 4	ATG/TAG	
tRNA-Glu	tRNA^Glu^	L	14,322–14,390	69	0		TTC
Cytochrome *b*	*cob*	H	14,395–15,535	1141	4	ATG/T	
tRNA-Thr	tRNA^Thr^	H	15,536–15,607	72	0		TGT
tRNA-Pro	tRNA^Pro^	L	15,607–15,676	70	‒ 1		TGG
Control region	CR	H	15,677–16,607	931	0		

^a^H and L refer to genes transcribed in the heavy and the light strand, respectively

^b^The number in the parenthesis indicates nucleotide base(s) of the intergenic spacer (positive number) or overlap (negative number)

**Table 2:** Nucleotide sequence characteristics of the mitogenomes of five genus *Gobiobotia* species including two *Gobiobotia naktongensis* populations from Geum and Nakdong Reivers in South Korea

Genus	Species	Populations	Accession number	Size (bp)	Whole mitogenome composition				
					A (%)	G (%)	T (%)	C (%)	A + T (%)
Gobiobotia	*G. naktongensis*	Geum River	in this study	16,607	30.3	16.8	26.6	26.3	56.9
	*G. naktongensis*	Nakdong River	KC353467	16,609	30.4	16.8	26.6	26.3	57.0
	*G. pappenheimi*	–	KU314697	16,605	30.2	16.9	26.6	26.3	56.8
	*G. brevibarba*	–	FJ515919	16,594	28.8	18.2	26.1	26.8	55.0
	*G. intermedia*	–	KF667523	16,608	27.6	19.1	26.1	27.2	53.7
	*G. macrocephala*	–	FJ515918	16,610	29.4	17.9	25.4	27.2	54.8

We compared the nucleotide composition of the mitogenomes of five *Gobiobotia* species, including two *G. naktongensis* populations (Table 2). The nucleotide composition of *G. naktongensis* Geum River was A = 30.3%, G = 16.8%, T = 26.3%, and C = 26.3%, showing a bias toward A + T (56.9%), similar to other *Gobiobotia* species, which is similar to the results of most fish mitogenomes (Wang et al. 2020; Yang et al. 2018).

**Fig. 2:**
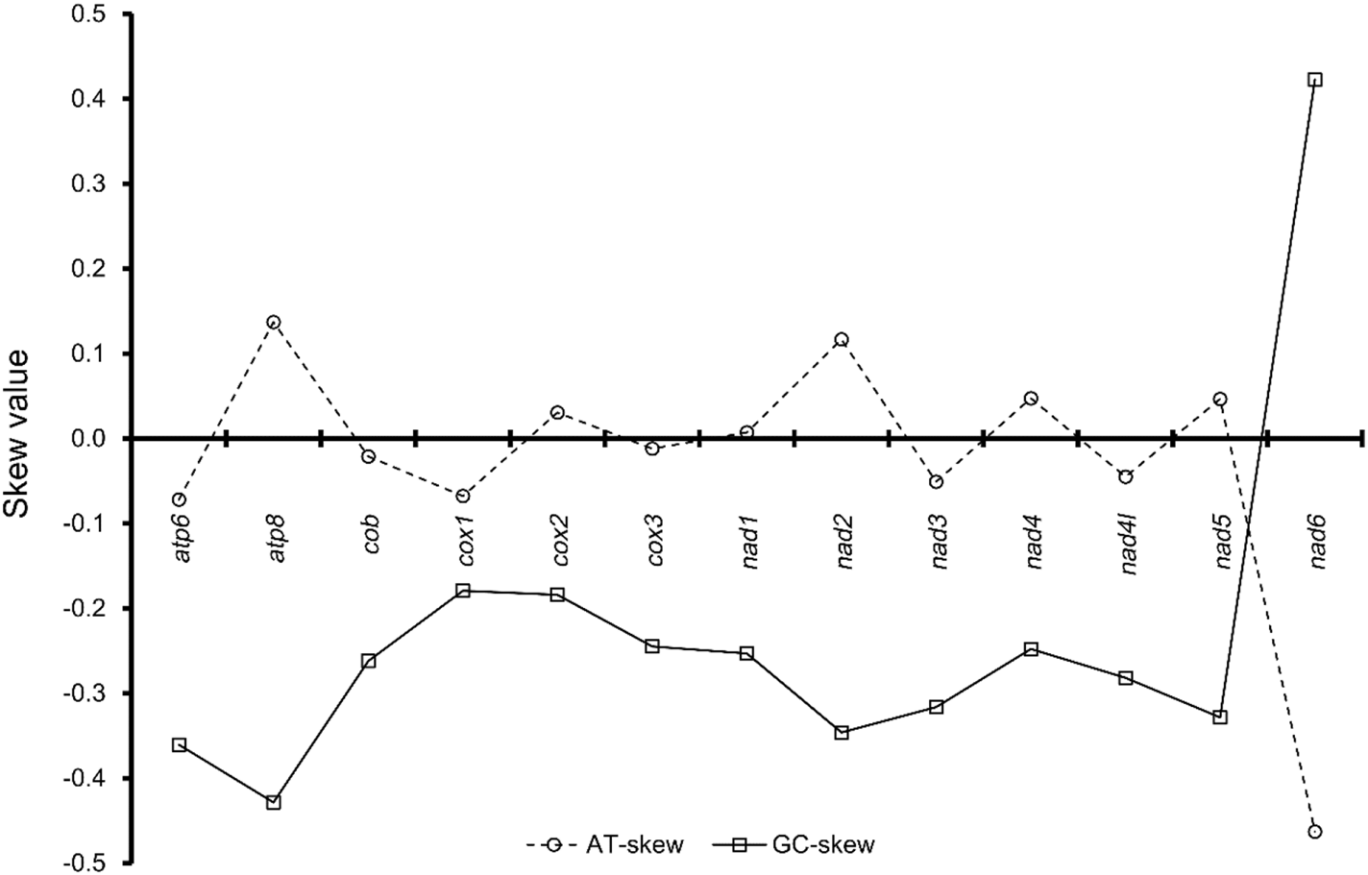
AT- and GC-skews of 13 protein-coding genes of the mitogenome of *Gobiobotia naktongensis* from the Geum River in South Korea

AT/GC skew is a method used to evaluate the excess of A and/or C nucleotides based on the H-strand; a positive skew value indicates that the T and/or G nucleotides consist of a relatively small number. Thus, AT/GC skew is a measure of compositional asymmetry. Owing to asymmetrical directional mutation pressure (Francino and Ochman 1997; Perna and Kocher 1995; Yang et al. 2018), such asymmetry is reflected in the codon usage of genes in different directions. For example, H-strand encoded genes show a clear preference for C in the codon wobble position, whereas L-strand encoded genes for G or T. The PCGs of the mitogenome of *G. naktongensis* Geum River had a slightly higher AT content (56.9%) than the rRNA genes (55.2%) (Table 3). The control region, which occupies most of the non-coding region, showed a significantly higher AT content (66.5%), similar to the mitochondrial genes of other fishes (Wang et al. 2020; Yang et al. 2018; Zhou et al. 2017). AT skews by gene regions ​​were all positive, except for PCGs, and all GC skews ​​were negative, except for tRNAs. Particularly, rRNA regions had a highly A-biased nucleotide composition, and 13 PCGs regions had a highly C-biased composition. The AT skews among the 13 PCGs in *G. naktongensis* Geum River waved near zero, ranging from ‒ 0.072 to 0.117, except for *nad6* (‒ 0.462), and the values of negative and positive AT skews were similar (Fig. 2). All GC skews in the 13 PCGs ranged from ‒ 0.428 to ‒ 0.179 except for *nad6* (0.423). This result suggests that more C nucleotides are present in most PCGs, and *nad6* only presented negative AT and positive GC skews, which is consistent with most previous reports of strand asymmetry in freshwater fish (Hwang et al. 2013b, c).

**Table 3:** Nucleotide sequence characteristics according to the functional groups of genes of the mitogenomes in *Gobiobotia naktongensis* from the Geum River in South Korea

Genes	Nucleotide frequency				A + T (%)	AT skew	GC skew
	A (%)	G (%)	T (%)	C (%)			
PCGs	28.0	16.6	28.6	26.8	56.9	− 0.010	− 0.235
rRNAs	34.2	21.1	21.0	23.7	55.2	0.239	− 0.058
tRNAs	29.5	22.7	27.1	20.8	56.6	0.043	0.044
Control region	33.3	13.6	33.2	19.9	66.5	0.002	− 0.186

**Fig. 3:**
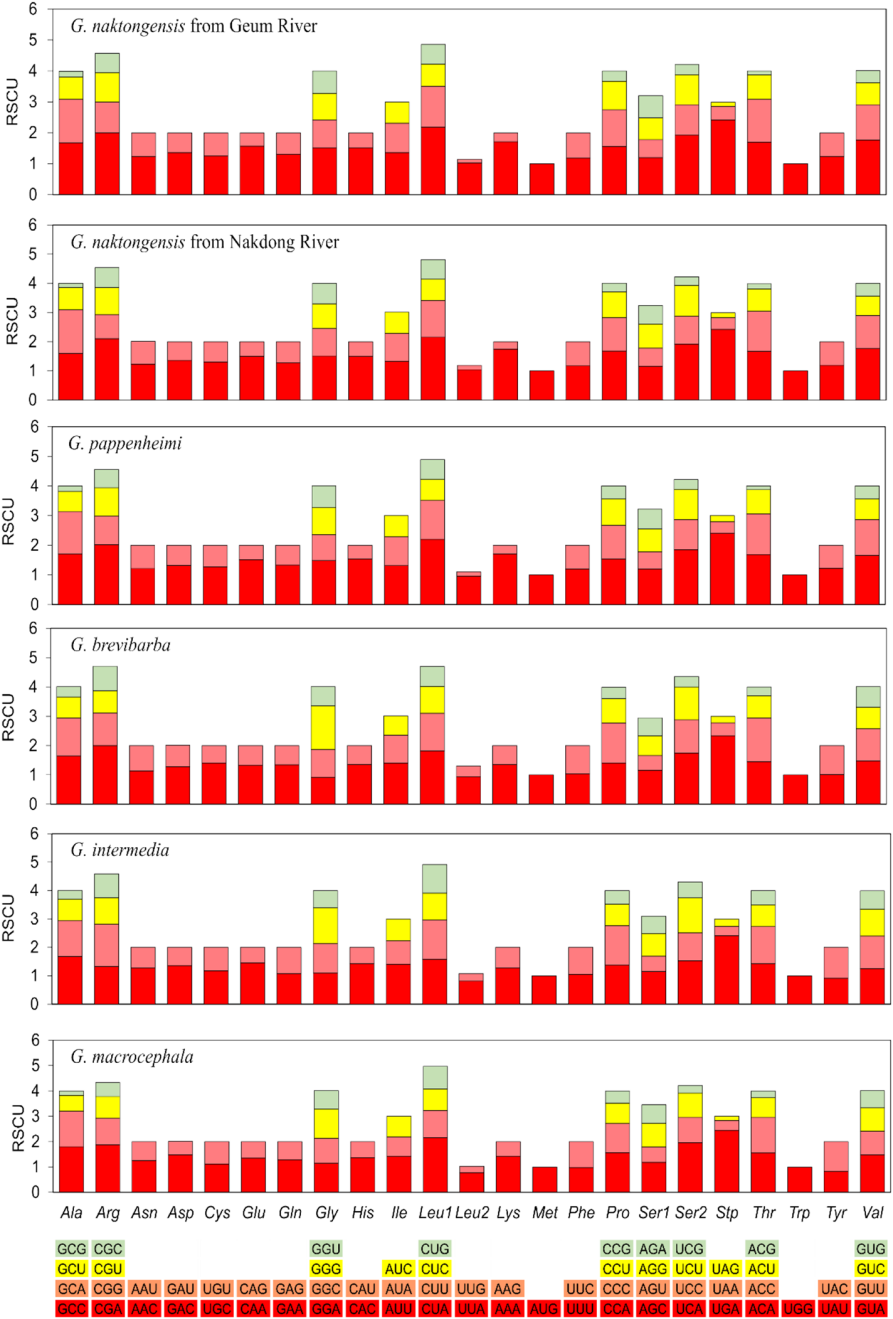
Relative synonymous codon usage (RSCU) of the 13 protein-coding genes in the mitogenome of genus *Gobiobotia* species

Among 3,857 codons encoded by 13 PCGs, the amino acids Ala, Arg, Gly, Leu1, Pro, Ser1, Ser2, Thr, and Val were utilized by four different codons, and the other amino acids were encoded by either one or three. Figure 3 shows the amino acid codon usage by relative synonymous codon usage (RSCU) values for the genus *Gobiobotia*. The result of RSCU analysis did not show any difference of codon type in genus *Gobiobotia*, and revealed that the codons encoding Leu1 (CUA), Arg (CGA), and Ser2 (UCA) were the most frequently present, while Leu2 (UUG) and Ala (GCG) were the least frequently present. The only exception was *G. intermedia*, in which the proportion of Ala (GCC) was the most frequently used codon. The A + T content and AT/GC skew ​​of the 13 PCGs are closely related to codon usage (Chao et al. 2014; Shi et al. 2016). The results of the RSCU analysis showed that the most frequently used codons at the 3rd position were A, and the least frequently used codons were G, indicating that A or C were used more frequently than T or G in codons at the 3rd position, indicating saturation (Yamanoue et al. 2007).

**Fig. 4:**
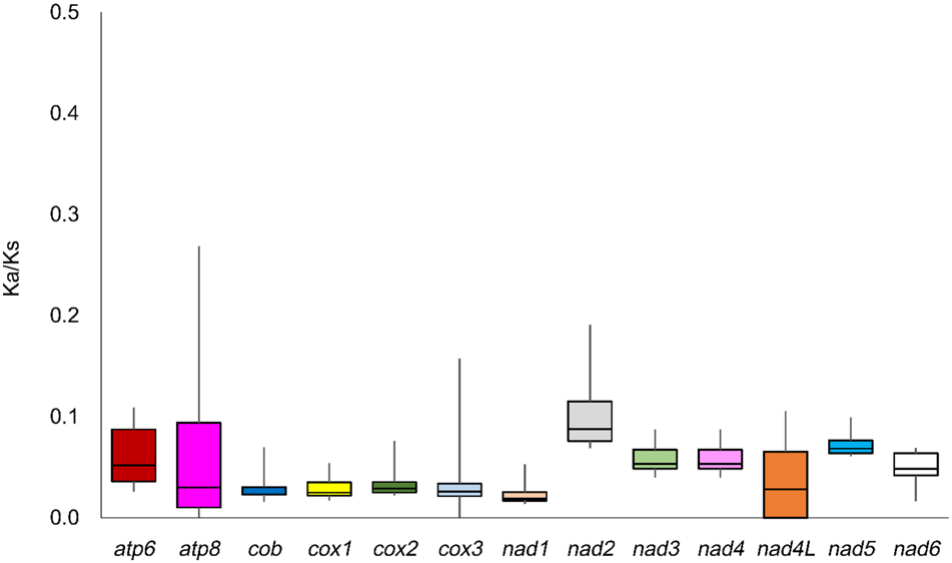
Comparisons of the average Ka/Ks ratios for 13 protein-coding genes among five *Gobiobotia naktongensis* species including two *Gobiobotia naktongensis* populations from the Geum and Nakdong Rivers in South Korea

The Ka (non-synonymous)/Ks (synonymous) ratio is particularly useful for determining the evolutionary relationship between PCGs in the mitogenomes of closely related species (Fay and Wu 2003). This ratio is an indicator of the selective pressure on PCGs: negative selection (Ka/Ks < 1), positive selection (Ka/Ks > 1), and the balance of both selections (Ka/Ks = 1) (Meganathan et al. 2011; Li et al. 2012). The Ka/Ks ratios of all PCGs among *Gobiobotia* species were < 1 (range 0.024–0.100), suggesting that they were under strong negative (purifying) selection and environmental changes were not large enough to change genetic function (Fig. 4). Additionally, no Ks sites (synonymous) were detected in *atp8* between *G. naktongensis* Geum River and *G. pappenheimi*.

The mitogenome of *G. naktongensis* Geum River contains 22 tRNA genes that are typically found in vertebrates. Their lengths ranged from 68 bp (tRNA^Cys^) to 76 bp (tRNA^Lys^). Most tRNAs, except for tRNA^Ser1^, were predicted to be folded into typical cloverleaf secondary structures, and like most vertebrates, tRNA^Ser1^ lacked a recognizable D-arm with a loop (Cui et al. 2007; Zhou et al. 2009). Twelve mismatched pairs (mainly A–C, and rarely U–U, A–A, C–C, and U–C) were predicted in nine genes (tRNA^Phe^, tRNA^Val^, tRNA^Met^, tRNA^Trp^, tRNA^Arg^, tRNA^His^, tRNA^Ser1^, tRNA^Thr^, and tRNA^Ser2^) among the 22 tRNA genes, and G was inserted at the 3′-end (upward direction) of the TΨC-stem of tRNA^Ser1^. The mismatch of the stem region can be corrected through a post-transcriptional RNA editing mechanism (Masta and Boore 2004).

**Fig. 5:**
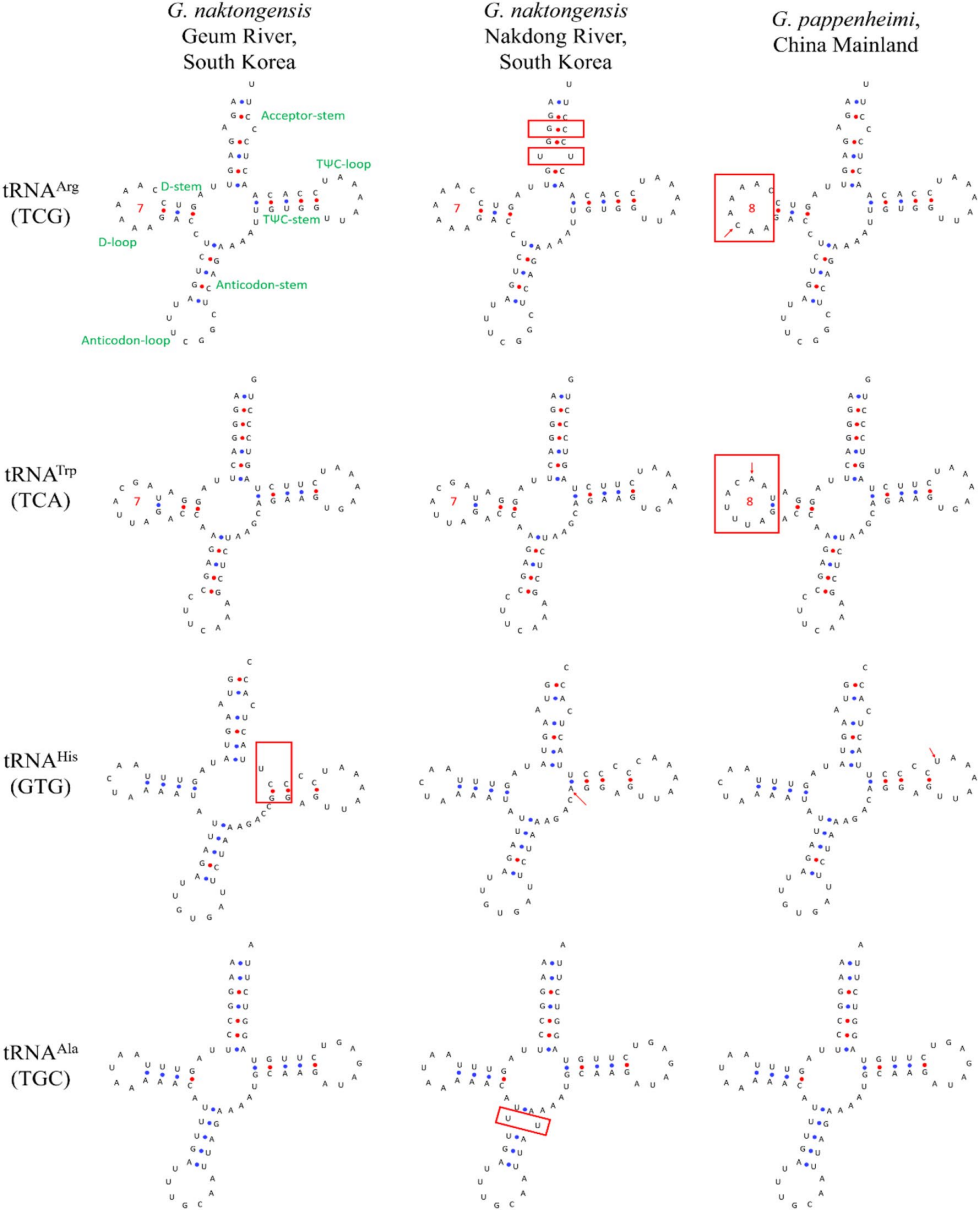
Comparison of tRNA secondary structures in the mitogenomes of two *Gobiobotia naktongensis* populations from the Geum and Nakdong Rivers in South Korea and *G. pappenheimi* in Mainland China. Red arrows indicate point mutations, and red boxes indicate structural changes. The numbers in the D-loop region indicate their lengths of nucleotide sequences

The prediction of the secondary structure of tRNA genes among *G. naktongensis* Geum and Nakdong Rivers and *G. pappenheimi* revealed 17 base substitutions (including indels) in 11 of 22 tRNA genes (data not shown). Among them, nine substitutions were specific to *G. naktongensis* Nakdong River, three specific to *G. naktongensis* Geum River, and the other five substitutions were specific to *G. pappenheimi*. tRNA genes showing structural differences are indicated in Fig. 5. In tRNA^Arg^ and tRNA^Ala^, structural differences in the acceptor and anticodon stems were found because of specific base substitutions in *G. naktongensis* Nakdong River. The D-loop showed differences in the lengths of the two *G. naktongensis* populations (7 bp) and *G. pappenheimi* (8 bp) due to the base indels of tRNA^Arp^ and tRNA^Trp^. Moreover, the lengths of tRNA^His^ were the same, but showed a length difference in the variable region between the anticodon-stem, TΨC-stems, and TΨC-loops. All tRNAs were more conserved than synonymous regions, except for the variable loops, and the TΨC- and D-stem regions were more conserved than the loop regions (Vilmi et al. 2005). Point mutations within the tRNA gene have the potential to affect the tRNA metabolic fate structurally, functionally (or both) critical positions for the tRNA metabolic fate (Helm et al. 2000). Therefore, *G. naktongensis* Geum and Nakdong Rivers may be taxonomically different from *G. pappenheimi*.

**Fig. 6:**
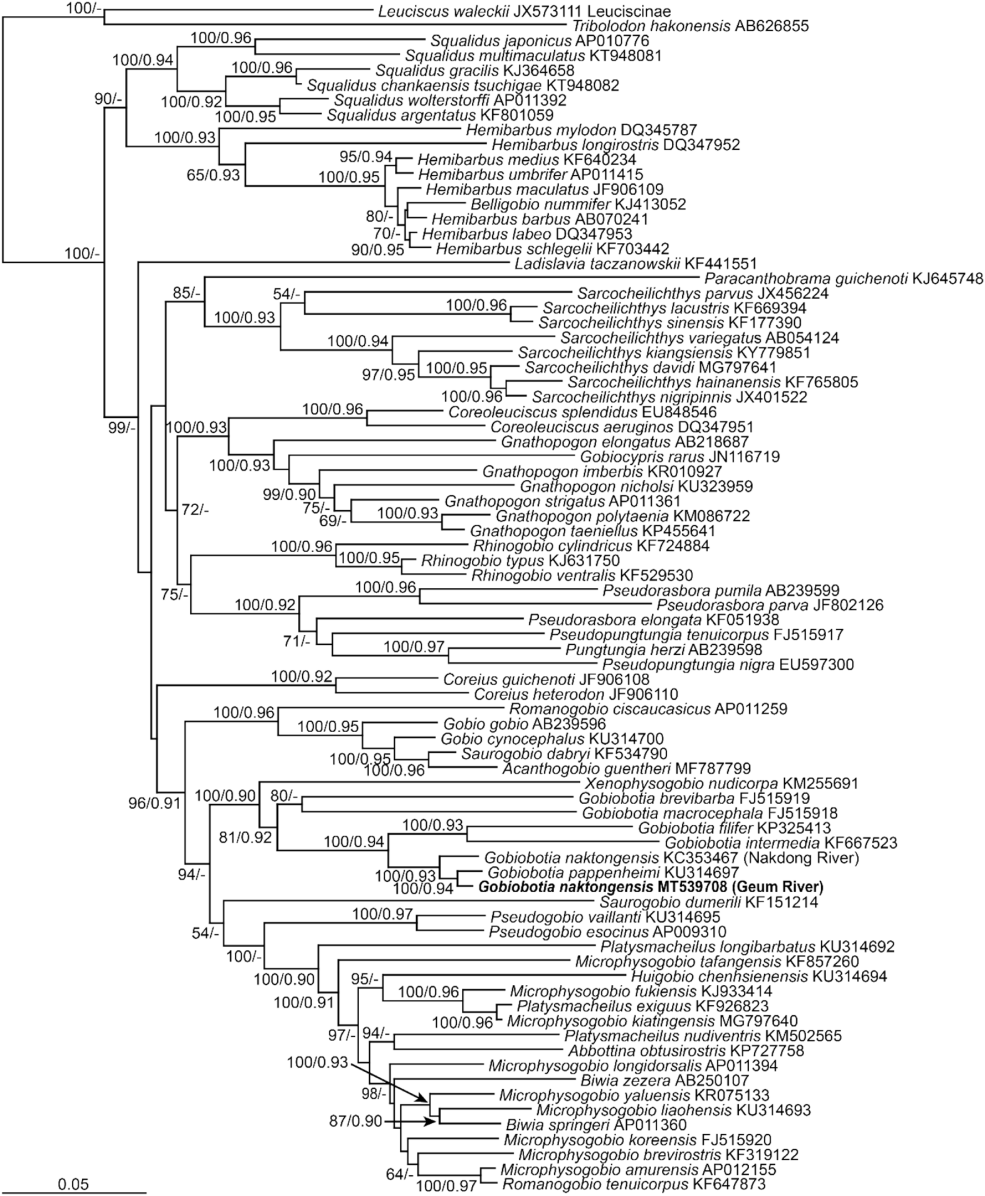
Maximum-likelihood (ML) phylogenetic tree based on the complete mitogenomes from gobionid species belonging to the order Cypriniformes. The nucleotide sequence matrix included the first and second codon positions of 12 protein-coding genes and unambiguously aligned regions of two ribosomal RNA and 22 transfer RNA genes. Bootstrap value above 50% in the ML analysis and posterior probability value above 0.90 in the Bayesian inference analysis are indicated at each node. *Gobiobotia naktongensis* analyzed in this study is shown in bold

Phylogenetic trees were reconstructed based on the BI and ML methods using the mitogenomic sequence matrix from 12 PCGs, 2 rRNA genes, and 22 tRNA genes of the representative gobionid species, including *G. naktongensis* Geum River. In the resulting trees, all the gobionid species formed a monophyletic group with respect to the two outgroups. All *Gobiobotia* species clustered with *Xenophysogobio nudicorpa* and were clearly distinct from the other gobionid genera (Fig. 6). Within the lineage, *G. naktongensis* Geum River showed a closer relationship to *G. pappenheimi* in mainland China with 100% bootstrap value in ML analysis and 0.94 posterior probability value in BI analysis, rather than its conspecific population reported from the Nakdong River, its type locality (Mori 1935).

Recent phylogenetic studies have revealed significant genetic differences in freshwater fish fauna between major river systems in South Korea (Kim and Bang 2012; Won et al. 2020). In South Korea, many independent rivers have developed because of the large number of mountain ranges, which is known to have occurred due to large-scale events between the Early Triassic and Early Miocene (Chough et al. 2000; Won et al. 2020). They have served as vicariant barriers that have shaped the current biogeography and allopatric speciation of fish assemblages in the Korean Peninsula (Fig. 1) (Kim et al. 2005). Previous phylogenetic studies of two *Coreoleuciscus* species (Kim et al. 2012) and two *Koreocobitis* species (Kim and Bang 2012), endemic to the Korean peninsula, suggested that their biogeography was clearly divided into two disjunct areas, the West Korea and South Korea subdistricts, by the major vicariant barriers along the Noryeong/Sobaek mountain ranges and Baekdudaegan mountain range. The two *G. naktongensis* populations from the two major river systems, the Geum and Nakdong rivers in the West Korea and South Korea subdistricts, respectively, also showed the same biogeographical distribution pattern as those of *Coreoleuciscus* and *Koreocobitis* (Kim and Bang 2012; Kim et al. 2012). Each of the two species in both genera was erected as a novel species based on further taxonomic studies, as well as significant genetic divergence (Kim et al. 2000; Song and Bang 2015).

According to Lindberg (1972) and Nishimura (1974), when sea levels fell during the Ice Age, the Han (including the Imjin River) and Geum River systems in the Korean Peninsula were connected to the Yellow River system (Huanghe) in mainland China (the Paleo-Huanghe River). Thus, our results showing a closer relationship of *G. naktongensis* Geum River to *G. pappenheimi* in mainland China than its conspecific population from the Nakdong River provides important evidence for the existence of the Paleo-Huanghe River system that had shaped the distinct fish assemblages in the West Korea Subdistrict, including the Han, Imjin, and Geum River systems.

The *G. naktongensis* populations of the Imjin, Han, and Geum Rivers should be taxonomically separated from the population of the Nakdong River, and their taxonomic status should be considered in future studies. In addition to taxonomic issues between the two *G. naktongensis* populations, the existence of the two distinct lineages strongly suggests that both should be protected because populations from separate biogeographical regions with significant genetic variation may be considered separate evolutionarily significant units.

## Conclusions

In this study, we analyzed the complete mitogenome of the *G. naktongensis* population from the Geum River to construct a phylogenetic tree of gobionid species. This study provides baseline data for the molecular identification, geographical distribution, and phylogenetic study of endangered freshwater fish species in the Korean peninsula. These will also be essential for establishing plans and strategies for the conservation and restoration of this evolutionarily significant unit.

## Data Availability

The sequence data were deposited in the nucleotide database [MT539708] (https://www.ncbi.nlm.nih.gov/nuccore/MT539708.1/).
